# Intact mitochondrial function in the setting of telomere‐induced senescence

**DOI:** 10.1111/acel.13941

**Published:** 2023-09-08

**Authors:** Daniel I. Sullivan, Fiona M. Bello, Agustin Gil Silva, Kevin M. Redding, Luca Giordano, Angela M. Hinchie, Kelly E. Loughridge, Ana L. Mora, Melanie Königshoff, Brett A. Kaufman, Michael J. Jurczak, Jonathan K. Alder

**Affiliations:** ^1^ Dorothy P. and Richard P. Simmons Center for Interstitial Lung Disease, Division of Pulmonary, Allergy, and Critical Care Medicine University of Pittsburgh Pittsburgh Pennsylvania USA; ^2^ Division of Endocrinology and Metabolism University of Pittsburgh Pittsburgh Pennsylvania USA; ^3^ Center for Metabolism and Mitochondrial Medicine University of Pittsburgh Pittsburgh Pennsylvania USA; ^4^ Heart, Lung, and Blood Vascular Medicine Institute University of Pittsburgh Pittsburgh Pennsylvania USA; ^5^ Division of Pulmonary, Critical Care and Sleep Medicine, Davis Heart Lung Research Institute The Ohio State University Columbus Ohio USA

**Keywords:** SASP, shelterin, telomerase

## Abstract

Mitochondria play essential roles in metabolic support and signaling within all cells. Congenital and acquired defects in mitochondria are responsible for several pathologies, including premature entrance to cellar senescence. Conversely, we examined the consequences of dysfunctional telomere‐driven cellular senescence on mitochondrial biogenesis and function. We drove senescence in vitro and in vivo by deleting the telomere‐binding protein TRF2 in fibroblasts and hepatocytes, respectively. Deletion of TRF2 led to a robust DNA damage response, global changes in transcription, and induction of cellular senescence. In vitro, senescent cells had significant increases in mitochondrial respiratory capacity driven by increased cellular and mitochondrial volume. Hepatocytes with dysfunctional telomeres maintained their mitochondrial respiratory capacity in vivo, whether measured in intact cells or purified mitochondria. Induction of senescence led to the upregulation of overlapping and distinct genes in fibroblasts and hepatocytes, but transcripts related to mitochondria were preserved. Our results support that mitochondrial function and activity are preserved in telomere dysfunction‐induced senescence, which may facilitate continued cellular functions.

## Graphical abstract

Cellular senescence brings about a constellation of changes within cells including altered cellular morphology, transcription, and secretory behavior. Sullivan et al. examined the consequences of telomere dysfunction on mitochondria in fibroblasts in vitro and on hepatocytes in vivo. They found that mitochondrial function was remarkably preserved during cellular senescence in vitro as well as in vivo.

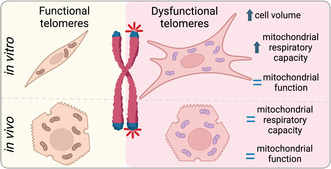

**Abbreviations**
4HT: 4‐hydroxytamoxifen
GSEA: gene set enrichment analysis
iMEF: immortalized murine embryonic fibroblast
NEFA: triglyceride, cholesterol, and free fatty acid
RCR: respiratory control ratio

## 1 INTRODUCTION

Cellular senescence is an irreversible exit from the cell cycle that is brought on by numerous forms of cellular stress and is hypothesized to play a role in many age‐associated diseases.

Senescence is thought to contribute to aging as stem‐progenitor cells lose their intrinsic capacity to proliferate. Senescent cells may also persist for long periods of time and contribute to tissue dysfunction by secreting pro‐inflammatory signals or altering the extracellular matrix (Coppé et al., 2010). Numerous past studies have documented substantial changes in cellular behavior upon entrance to cellular senescence. Notable changes include an enlarged and flattened morphology, expanded lysosomal activity with increased expression of senescence‐associated beta‐galactosidase (SA‐βgal), altered gene expression, modified secretory behavior, and decreased metabolic and mitochondrial activity (Coppé et al., 2010; Dimri et al., 1995; Hayflick & Moorhead, 1961; Hernandez‐Segura et al., 2017; Mortimer & Johnston, 1959; Sahin et al., 2011).

Several forms of cellular stress have been reported to drive cellular senescence. The most widely studied mechanism is replicative senescence brought on by telomere shortening. Telomeres are repetitive DNA sequences that are found on the ends of chromosomes and become progressively shorter in somatic cells with each cellular division. Telomeres bind a set of six proteins, known collectively as the shelterin complex, that function to suppress the DNA damage response. Within the shelterin complex, telomeric repeat binding factor 2 (TRF2) binds to telomere sequences and suppresses the DNA double‐strand break response (de Lange, 2018; Okamoto et al., 2013). Despite shelterin's protective role, recurrent rounds of DNA replication eventually lead to critical telomere shortening and the induction of a DNA damage response. This halts the cell cycle via expression of cell cycle inhibitors, including *CDKN1A* (p21), among others (d'Adda di Fagagna et al., 2003). Telomere dysfunction can be triggered in a uniform fashion by conditional deletion of *Terf2*, encoding TRF2, and has been used to study cellular senescence in a cell type specific manner (Alder et al., 2015; Lazzerini Denchi et al., 2006). In addition to telomere shortening, DNA damage, oncogene activation, and mitochondrial dysfunction have all been reported to drive senescence (Bartkova et al., 2006; Di Micco et al., 2006; Wiley et al., 2016).

While mitochondrial dysfunction can drive cellular senescence (Gallage & Gil, 2016; Wiley et al., 2016), several reports documented mitochondrial dysfunction as part of the senescence phenotype. For example, during replicative senescence in primary human fibroblasts, although bulk mitochondrial respiratory capacity was unaffected, a subpopulation of senescent cells displayed partial uncoupling, which was associated with increased oxidative stress (Hutter et al., 2004). Furthermore, mitochondrial dysfunction in senescent cells has been reported to be responsible for the heterogenous response to telomere dysfunction and to mediate a feed‐forward loop of DNA damage and telomere shortening via the generation of reactive oxygen species (Passos et al., 2007). Additionally, senescence‐associated changes in mitochondrial activity were reported in late‐generation telomerase RNA component (*Terc*; also known as TR) knockout mice and resulted in decreased mitochondrial biogenesis due to downregulation of *Ppargc1a* in vivo. This was partially mediated by p53, but a p53‐independent mechanism also functioned to suppress mitochondrial activity in cells with telomere‐mediated senescence (Sahin et al., 2011). Despite these findings, the interactions between telomere dysfunction and mitochondrial dysfunction remain incompletely understood.

Prior studies examining the consequences of telomere dysfunction on mitochondrial activity were limited by the fact that they were performed in the setting of replicative senescence, where the entrance to cellular senescence is asychronous and heterogeneous. Moreover, in vivo studies were performed in short telomere mice where telomere lengths are extremely heterogeneous and paracrine or systemic factors could influence mitochondrial function. We, therefore, undertook detailed molecular characterization of the consequences of telomere dysfunction on mitochondrial function using a tractable system that permits the induction of synchronous and uniform senescence. Our results suggest that mitochondrial function is remarkably preserved in the context of telomere dysfunction‐mediated senescence.

## 2 METHODS

### 2.1 Generation of murine lines

The following mice were obtained from The Jackson Laboratory: B6.129P2‐*Terf2* ^*tm1Tdl*^ */J* (hereafter referred to as *Trf2* ^*F/F*^), B6.129‐*Gt(Rosa)26Sor* ^*tm1(cre/ERT2)Tyj*^ */J* (hereafter referred to as *CreER*), and B6.129(Cg)‐*Gt(ROSA)26Sor* ^*tm4(ACTB‐tdTomato,‐EGFP)Luo*^/J (hereafter referred to as Rosa^mTmG/mTmG^). All strains were on a C57BL/6J background. *Trf2* ^*F/F*^ mice were bred with *CreER* mice to generate *Trf2* ^*F/F*^;*CreER* mice to generate mouse embryonic fibroblasts. *Trf2* ^*F/F*^ mice were bred with Rosa^mTmG/mTmG^ mice to generate Trf2^F/F^;Rosa^mTmG/mTmG^ mice. All mice were housed under standard conditions at the University of Pittsburgh and experiments were approved by the Institutional Animal Care and Use Committee.

### 2.2 Western blots

Western blots were performed following standard procedures and employed antibodies specific for TRF2 (Novus Biologicals, NB110‐57130), RAP1 (Cell Signaling, D9H4), Lamin B1 (Santa Cruz Biotechnology, sc‐374,015), and Rhodamine Anti‐GAPDH (BioRad). Briefly, cells were lysed in RIPA buffer containing protease and phosphatase inhibitors (MiniComplete, Roche). Following protein quantitation, 20–40 μg of protein were separated under reducing conditions using SDS‐PAGE and transferred to PVDF membranes. Proteins were blotted with antibodies specific to the desired protein and visualized on a ChemiDoc MP gel documentation system (BioRad). Mitochondrial electron transport chain proteins were identified using the antibody mix and instructions included in the OxPhos Rodent WB Antibody Cocktail (ThermoFisher) according to the manufacturer's protocol.

### 2.3 Isolation of mouse embryonic fibroblasts (MEFs)

MEFs were isolated from mouse embryos derived from *Trf2* ^*F/F*^ and *Trf2* ^*F/F*^;*CreER* mothers at 12 days of gestation. Briefly, the visceral organs of each embryo were removed, and the remaining tissue was minced and incubated in 3 mL 0.25% trypsin (Gibco) per embryo and incubated at 37°C for 30 min. Trypsin was inactivated by the addition of DMEM (Gibco) containing 10% FBS (Gibco) and cells were then plated in 10 cm culture dishes and allowed to adhere for 24 h. Nonadherent cells were then discarded and cells in the adherent fraction were termed MEFs. Early passage cells were immortalized by transduction with pBABE‐zeo largeTgenomic (a gift from Bob Weinberg; Addgene plasmid # 1778). Following transduction, cells were selected with zeocin (ThermoFisher) and then cryopreserved. Hereafter, these immortalized MEFs will be referred to as iMEFs.

### 2.4 Tissue culture and cell line characterization

iMEF cells were cultured in DMEM supplemented with 10% fetal bovine serum and penicillin (120 U/mL), streptomycin (100 mcg/mL), and L‐glutamine (2 mM). Proliferation studies were carried out by plating three independent cultures of each cell line and enumerating cells at each passage. The total number of cells was log2 transformed and plotted against time. Fresh 4‐hydroxytamoxifen (4HT; 1 μM final concentration) was added at each passage and every 48 h if not due for passaging at that time. Clonogenic assays were performed by plating 1000 cells in 10 cm dishes and enumerating colonies following staining with crystal violet after 12 days in culture. A BioRad ChemiDoc™ MP Imaging System was used for image acquisition of clonogenic assays. Senescence‐associated beta galactose staining was performed on iMEFs 7 days after the addition of 4HT (Cell Signaling, #9860).

### 2.5 Transcriptional profiling and analysis

Total RNA was isolated from biological replicates (*n* = 3/group for iMEFs and *n* = 4/group for primary liver samples) using RNAeasy kits (Qiagen) according to the manufacturer's protocol and sent for library preparation, sequencing, quality control, and alignment at Novogene. Approximately 20 million paired‐end fragments were sequenced for each sample. Raw count tables from Novogene were analyzed using DeSeq2 (Love et al., 2014) for differential expression analysis and generating volcano plots in R. The raw data are available at NCBI's Gene Expression Omnibus (Edgar et al., 2002) GSE226613. Expression data from human cells entering replicative senescence was obtained from GSE175533 (Chan et al., 2022). Bulk RNA‐sequencing (RNA‐seq) data from population doublings <30 was used as control (*n* = 9) and compared to cells entering replicative senescence that had undergone >50 population doublings (*n* = 9). Additional analyses were conducted using GSEA (Mootha et al., 2003; Saul et al., 2022; Subramanian et al., 2005). Differential expression of several genes was confirmed using quantitative real‐time PCR with primers specific for the selected genes (PrimeTime qPCR Primer Assays, Integrated DNA Technologies). The expression levels of mitochondrial‐related genes (both nuclear and mitochondrial encoded) were analyzed using the MitoCarta 3.0 gene set (Rath et al., 2021).

### 2.6 iMEF immunostaining and imaging

Cells were grown in the presence of 1uM 4HT for 5 days and then plated on coverslips, where they grew for 2 days prior to being fixed in 2% PFA for 15 min. Following fixation, cells were washed, permeabilized with 0.1% Triton X‐100 in PBS, and blocked with 5% goat serum. Coverslips were incubated with primary antibodies, including mouse anti‐ATP5B (Invitrogen, MA1‐930). Proteins were visualized with secondary antibodies conjugated to Alexa Fluor 594 (Invitrogen, #A‐11005). Nuclei were stained with 4,6‐diamidino‐2‐phenylindole (DAPI). Photomicrographs of immunostained cells were obtained using a Nikon ECLIPSE NiE fluorescent microscope equipped with an ORCA‐FLASH 4.0 camera. Phase contrast photomicrographs were captured on a Nikon ECLIPSE Ts2 equipped with a Nikon DS‐Fi3 camera. Analysis of mitochondrial content and characteristics were performed in triplicate. Z‐stack images with a 0.3um step size were taken using the 60× oil objective. Z‐stacks were then deconvoluted, and a custom Nikon Elements G3 program was developed to measure mitochondrial characteristics. In brief, *z*‐stacks were aligned, mitochondrial boundaries were identified, and the background was removed. Nuclear boundaries were likewise established.

### 2.7 iMEF metaphase spread and FISH protocol

Metaphases were conducted as described below and as previously published (Cesare et al., 2015). In brief, when iMEFs reached ~50% confluency, they were treated with 0.1 μg/mL colcemid (KaryoMAX colcemid, Gibco) for 2 h to arrest cells in metaphase. Cells were then trypsinized and collected in a 15 mL falcon tube and treated with a 75 mM KCl hypotonic solution for 8–10 min at 37°C. They were then placed on ice, and an ice‐cold 3:1 methanol to glacial acetic acid solution fixative was added to a total of 10% v/v fixative to hypotonic solution. Cells were centrifuged and then resuspended in cold fixative, prior to centrifuging again. The suspended cells were dropped from a height of approximately 3 feet onto slides that had been soaked in cold methanol and dipped in ice water. The slides were streamed for approximately 3 s before incubating on a wet paper towel for 5 min and then allowed to dry overnight. Slides were checked under a light microscope to ensure metaphase spreading before continuing. Slides were placed in EasyDip™ Slide Staining Jars and rehydrated for 5 min in PBS at 37°C, fixed with 2% PFA at room temperature for 5 min, washed 3× in PBS, and then treated with 100 μg/mL RNaseA (Qiagen) in PBS at 37°C for 15 min. Slides were then treated with 0.2 mg/mL pepsin (MP Biomedicals) in a 0.1 M HCl solution at 37°C for 15 min, fixed again with 2% PFA at room temperature for 5 min, and then washed 3× with PBS. Slides were dehydrated using a 70–90‐100 ethanol grade and allowed to dry to completion. A TTAGGG PNA probe was synthesized at PNABio. The PNA probe was constructed of Cy3 conjugated (TelC‐Cy3, F1002) repeats of 5′‐CCCTAA‐3′. The TTAGGG PNA probe was prepared at a concentration of 0.5 μg/mL in a PNA hybridization solution (70% formamide [v/v], 0.25% [w/v] blocking reagent, 10 mM Tris‐Cl, pH 7.5). 40 μL of hybridization solution was pipetted onto the slides, and a glass coverslip was placed on top before heating to 83°C for 5 min. Slides were then incubated in a humid chamber overnight at 4°C. The following morning, slides were washed twice for 15 min/wash with PNA wash A solution (70% [v/v] deionized formamide and 10 mM Tris‐Cl, pH 7.5) and then three times for 5 min/wash with PNA wash B solution (50 mM Tris‐Cl, pH 7.5, 150 mM NaCl, 0.1% [v/v] Tween 20) with DAPI added at a concentration of 500 ng/mL to the final 5 min wash. Slides were again dehydrated using a 70–90‐100 ethanol grade and allowed to dry to completion before mounting with ProLong™ Diamond Antifade Mountant. Slides were imaged at 60× on a Nikon ECLIPSE Ni fluorescent microscope.

### 2.8 Mitochondrial to nuclear DNA ratio

Total DNA was extracted from snap‐frozen cells, that were resuspended in proteinase K buffer (100 mM Tris–HCl pH 8.5, 5 mM EDTA, 0.2% SDS, 200 mM NaCl), 0.6 mg/mL proteinase K (Fisher Scientific) and 0.01% 2‐mercaptoethanol, and lysed overnight at 55°C. Proteins and cell membranes were precipitated by adding NaCl (1. 27 M) followed by centrifugation at 28,000 RCF for 15 min at 4°C. The supernatant was collected and mixed with 100% ethanol. The nucleic acids were isolated by centrifugation at 28,000 RCF for 15 min at 4°C. The pellet was washed with 70% ethanol, followed by another centrifugation at 28,000 RCF for 10 min at 4°C, and air‐dried overnight. DNA was resuspended in DNase‐free water and 0.4 mg/mL RNase A (Sigma‐Aldrich) for 2 h at 37°C to degrade RNAs. DNA was stored at −20°C and successively quantified by AccuBlue Broad Range dsDNA Quantitation kit (Biotium).

Mitochondrial DNA (mtDNA) and nuclear DNA (nDNA) were measured by duplex qPCR using TaqMan probes in a 96‐well StepOnePlus™ Real‐Time PCR System (Applied Biosystems, Thermo Fisher Scientific). The reaction contained a DNA template, Luna Universal qPCR Master Mix (New England Biolabs), and two primer‐probes sets for mtDNA (*Cox1*) and nDNA (*B2m*), respectively. Thermocycling conditions were as follows: initial denaturation step at 95°C for 20 s, 40 cycles, each consisting of denaturation at 95°C for 1 s, annealing at 62°C for 20 s, and elongation at 60°C for 20 s. Mitochondrial DNA was normalized to the nDNA by the ΔCt method for each sample. The mtDNA/nDNA in the groups were calculated as the variation of the control group by a 2^−ΔΔCt^ method.

### 2.9 Murine transduction

Rosa^mTmG/mTmG^ and Trf2^F/F^;Rosa^mTmG/mTmG^ mice were transduced with 1 × 10^12^ genomic copies of a custom AAV8 virus that expressed Cre‐recombinase controlled by the *TBG* promoter via tail vein injection. Following transduction, mice were monitored for 6–7 weeks before collecting tissues. To evaluate the transduction efficiency, a portion of the liver was fixed in 4% PFA, cryoprotected in sucrose, and embedded in OCT. Frozen sections were evaluated as described above. Transduction efficiency was evaluated by measuring the proportion of hepatocytes that were GFP‐positive.

### 2.10 Plasma lipid measurement

Whole blood was collected in heparin‐coated syringes by cardiac puncture following euthanasia, and plasma was isolated following centrifugation to pellet red blood cells. Plasma was used to measure triglycerides (Thermo Fisher Infinity Triglyceride Reagent), total cholesterol (Wako Diagnostics Cholesterol E), and nonesterified fatty acids (Wako Diagnostics NEFA‐HR Kit) using the noted commercially available kits according to the manufacturer's instructions.

### 2.11 Biochemical analyses

Citrate synthase activity was determined as previously described (Kuznetsov *Analytical Biochemistry* 2002). Briefly, citrate synthase activity was measured by mixing the mitochondrially enriched fraction with citrate synthase activity buffer (0.25% Triton X‐100, 0.31 mM acetyl‐CoA, 0.1 mM 5,5′‐dithiobis [2‐nitrobenzoic acid], 0.5 mM triethanolamine, 5 μM EDTA, and 10 mM Tris–HCl), followed by addition of 5 mM of oxaloacetate to initiate the reaction. The change in absorbance at 412 nm was recorded every 10 s over a 2‐min period using a spectrophotometer set to 37°C, and activity was calculated as the slope from the linear portion of the curve and normalized to the protein concentration determined by BCA protein assay.

### 2.12 Mitochondrial preparations and functional assessments

For studies employing iMEFs, cells were chemically permeabilized by the addition of saponin (25 μg/mL) prior to the assessment of mitochondrial respiratory function. The concentration of saponin used was sufficient to permeabilize the cell membrane without affecting mitochondrial membrane activity, which we confirmed by the addition of cytochrome c during the respirometry protocols. A separate fraction of cells was retained for citrate synthase activity measures, described above. For mouse liver studies, mitochondrially enriched lysates were prepared by homogenizing fresh liver in SMET buffer (70 mM sucrose, 220 mM mannitol, 1 mM EDTA, and 10 mM Tris–HCl pH 7.5, and 0.25% BSA) with a glass vessel and Teflon pestle, followed by centrifugation (800× g, 10 min, 4°C) and collection of the supernatant. A portion of the supernatant was used for respirometry and citrate synthase activity, and the remainder was used to isolate mitochondria by centrifugation (8000× g, 10 min, 4°C). Mitochondrial respiratory capacity was assessed using an Oroboros O_2_K High‐Resolution Respirometer in MiR05 buffer at 37°C under constant mixing in a sealed, 2‐ml chamber. Respirometry assays were performed using sequential additions of substrates followed by ADP and antimycin A to determine state 4, state 3, and nonmitochondrial respiration. Substrate and inhibitor concentrations were as follows: pyruvate (5 mM), malate (2 mM), and glutamate (10 mM); adenosine diphosphate (ADP, 2 mM); palmitoylcarnitine (25 μM); and antimycin A (2.5 μM). Protein concentration of the mitochondrially enriched lysate and purified mitochondria was determined by BCA protein assay. The respiratory control ratio (RCR) was calculated as the ratio of state 3 (ADP‐stimulated respiration) relative to state 4 (respiration prior to the addition of ADP). Respiration in the presence of antimycin A was subtracted from state 3 and 4 respiratory rates to account for nonmitochondrial respiration.

## 3 RESULTS

### 3.1 Telomere‐induced cellular senescence drives transcriptional changes while mitochondrial biogenesis genes are spared

**FIGURE 1:**
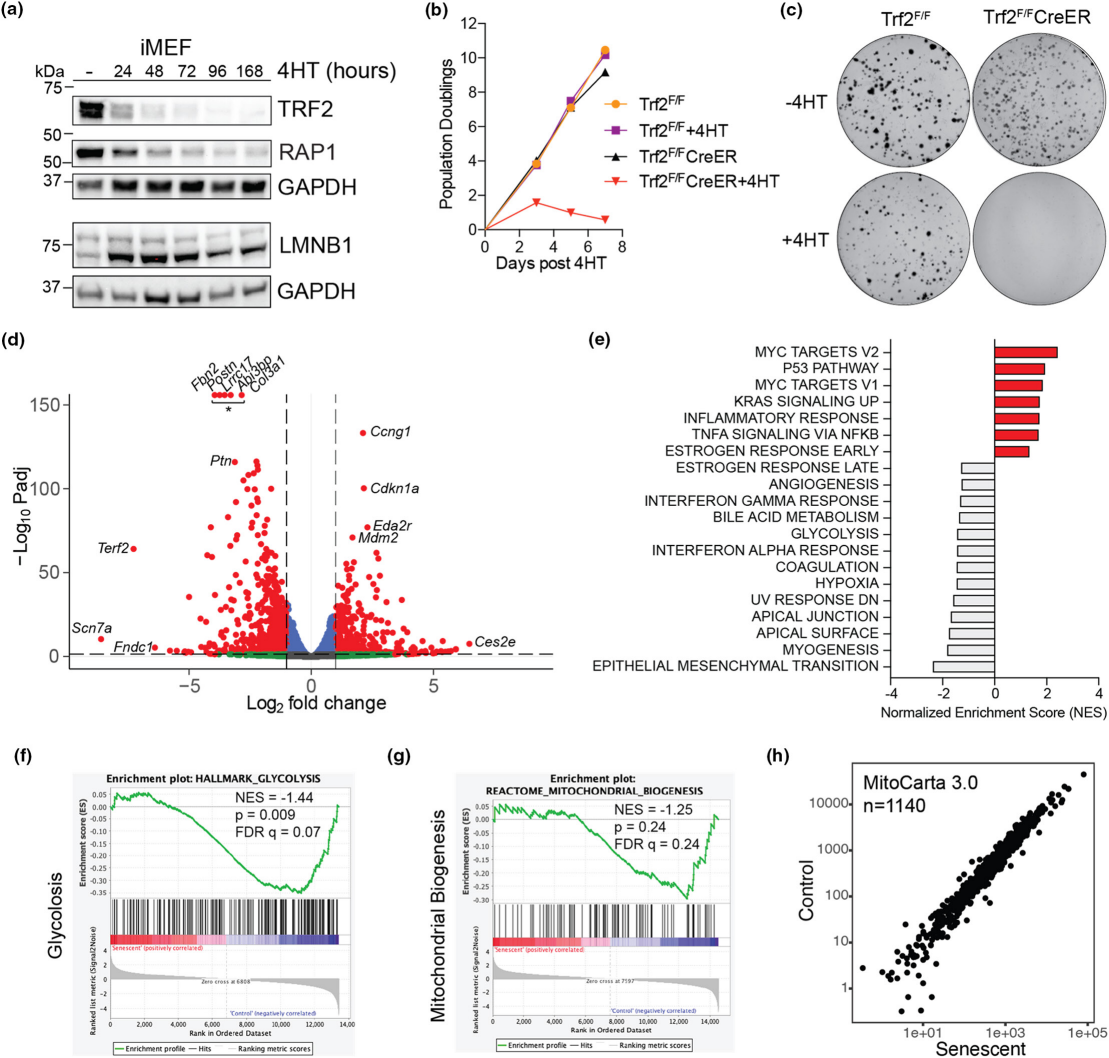
Induction of senescence causes global changes in gene expression but spares the mitochondrial transcriptome in a murine fibroblast cell line. (a) Time course of protein expression following induction with 4HT. Relative protein levels of TRF2, RAP1, LMNB1, and GAPDH (load control) are shown. (b) Relative proliferation of control groups (Trf2^F/F^, Trf2^F/F^ + 4HT, and Trf2^F/F^CreER) and senescent iMEFs (Trf2^F/F^CreER + 4HT). Viable cells were counted with trypan blue staining following induction with 4HT. Mean and standard error of the mean are shown for each count (*n* = 3). (c) Representative images of crystal violet stained clonogenic assays. Media was changed every 2–3 days after plating 1000 cells, and plates were imaged after 12 days. (d) Volcano plot depicting 5072 differentially expressed genes identified using RNA‐Seq of Trf2^F/F^CreER and Trf2^F/F^CreER + 4HT 7 days after induction (*n* = 3 per group). Notable genes are labeled. (e) Gene set enrichment analysis (GSEA) shows p53 signaling, inflammation, bile acid metabolism, glycolysis, and several pathways related to cell morphology as the most altered. (f) Enrichment plot for GSEA Hallmark Glycolysis Pathway. (g) Enrichment plot for Reactome Mitochondrial Biogenesis Pathway. (h) Differential expression of MitoCarta 3.0 genes showing similar expression in control and senescent cells. Normalized counts from DESeq2 are shown.

We sought to interrogate the consequences of dysfunctional telomere‐induced cellular senescence on mitochondrial homeostasis in a uniform fashion. To more fully characterize the mitochondrial sequela of telomere‐mediated senescence, we established an in vitro model of conditional telomere dysfunction. We generated SV40‐immortalized murine embryonic fibroblasts (iMEFs) from mice with conditionally removable alleles of the shelterin component TRF2 (Celli & de Lange, 2005) that had been bred with mice that expressed CreERT2 ubiquitously (*Terf2* ^*F/F*^;*Rosa26‐CreER* ^*T2*^, Trf2^F/F^;Rosa‐CreER hereafter). Before 4‐hydroxytamoxifen (4HT) was added to the media, Trf2^F/F^;Rosa‐CreER iMEFs grew normally and were indistinguishable from control Trf2^F/F^ iMEFs that did not express CreER. Upon addition of 4HT to the media, protein levels of TRF2 and its binding partner RAP1 decreased, and cells ceased proliferating (Figure 1a,b). Cells lacking TRF2 adopted characteristics of senescent cells including enlarged and flatted morphology, expression of senescence‐associated beta‐galactosidase, and a modest decrease in LMNB1 expression (Figure 1a and Figure S1a). Analysis of metaphase spreads from control and Trf2^F/F^;Rosa‐CreER iMEFs (Figure S1b) showed expected fusions of chromosomes in cells lacking TRF2 (van Steensel et al., 1998). Induction of senescence was uniform and complete with no cells escaping to form colonies after 12 days in culture (Figure 1c).

We next examined if genes associated with mitochondrial function or biogenesis were differentially expressed in cells following the induction of senescence. Deletion of TRF2 and the subsequent induction of senescence drove widespread changes in gene expression with more than 20% of the annotated genes showing statistically significant changes in gene expression (*n* = 5072, Padj <0.05; Figure 1d). *Cdkn1a*, the gene encoding p21 and an established marker of senescence, was one of the top upregulated genes. Gene set enrichment analysis (GSEA) of the SenMayo gene set showed a significant enrichment of genes associated with cellular senescence (Figure S1c) (Saul et al., 2022). Analysis of the GSEA hallmark pathways showed that p53 signaling and inflammation were significantly upregulated in senescent cells; whereas, pathways related to bile acid metabolism, glycolysis, and several pathways related to cell morphology were downregulated (Figure 1e,f and Figure S1d). We next investigated affected gene sets more closely to examine if specific pathways related to mitochondrial biogenesis had been altered. While there was a trend toward downregulation of genes related to mitochondrial biogenesis, this did not reach statistical significance (Figure 1g). We further evaluated the mitochondria‐associated genes that were previously reported to be downregulated in response to short telomeres (Sahin et al., 2011) and found no significant changes (Figure S1e). We reasoned that this may be due to SV40‐immortalization of MEFs and inactivation of the p53 signaling pathway, but found that this pathway was still engaged (Figure S1d), likely owing to the strong DNA damage signal induced by deletion of TRF2 (Alder et al., 2015). To comprehensively explore the changes in mitochondrial‐related genes, we examined the changes in 1140 genes related to mitochondrial function (MitoCarta 3.0) (Rath et al., 2021) in control and senescent cells and found that they were predominantly unchanged in senescence (Figure 1h). Unexpectedly, we found that all 13 transcripts encoded by the mitochondrial genome trended toward higher expression in senescent cells (Figure S1f). Finally, we tested if our findings were specific to cells with telomere dysfunction due to TRF2 deletion or if they could be extended to cells undergoing replicative senescence. We examined a large RNA‐seq data set of human fibroblasts undergoing replicative senescence, focusing on the expression of a curated list of 1136 mitochondrial‐associated genes (MitoCarta 3.0) in early‐ versus late‐passage cells and found their expression to be generally unchanged with a few exceptions (Figure S1h) (Chan et al., 2022). Together, these data suggest that mitochondrial‐related gene expression remains largely intact in the setting of telomere dysfunction‐induced cellular senescence.

### 3.2 Cellular senescence increases cell size and mitochondrial number in vitro

**FIGURE 2:**
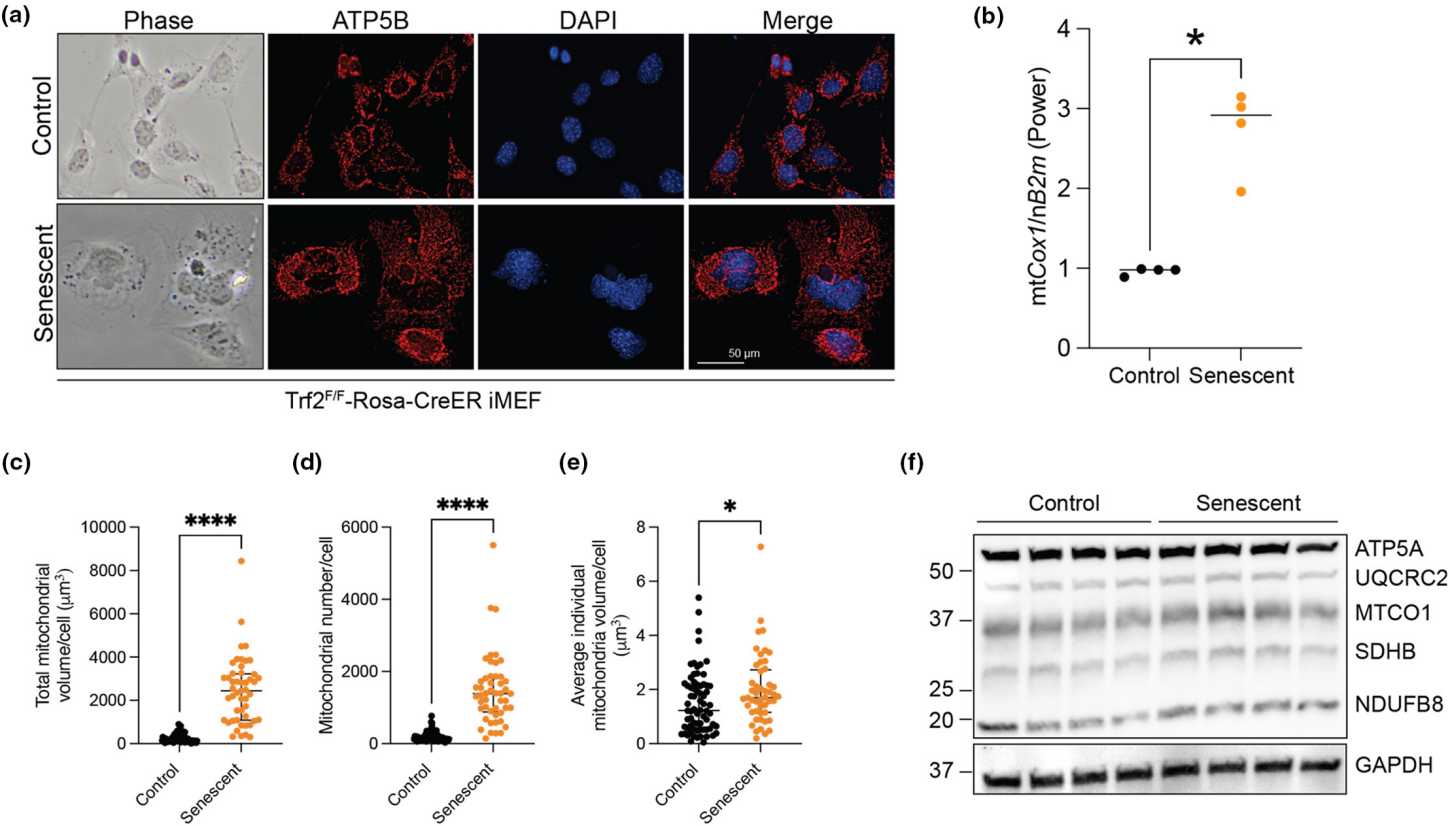
Cellular senescence drives changes in cellular morphology and mitochondrial composition. (a) Photomicrographs of control and senescent Trf2^F/F^CreER iMEFs using phase contrast (column 1) and immunofluorescence microscopy (columns 2–4). Images were taken 7 days after induction of Trf2^F/F^CreER with 4HT. (b) Mitochondrial:nuclear DNA ratio for control versus senescent iMEFs. (c) Total mitochondrial volume/cell (μm^3^). (d) Mitochondrial number/cell. (e) Average individual mitochondria volume/cell (μm^3^). (f) Western blot of mitochondrial electron transport chain components. *N* = 70 control and 49 senescent cells for (c–e); mean ± SD is shown. *p < 0.05 and ****p < 0.0001, Mann–Whitney test.

Changes in cellular morphology are a cardinal characteristic of cellular senescence in vitro. Indeed, following the deletion of TRF2, iMEFs underwent dramatic changes in cellular morphology, and enlarged, flattened cells became abundant (Figure S1a and Figure 2a). We next examined the mitochondria in these cells by staining for the mitochondrial inner membrane ATP synthase, ATP5B (Figure 2a). Senescent cells had more mitochondrial and nuclear material per cell, with mitochondria extending toward the edges of the cell membrane in both groups. The stark differences in nuclear and mitochondrial staining led us to examine the mitochondrial DNA copy number (mitochondrial: nuclear DNA ratios) in these cells, where we found a 3‐fold increase in the senescent cells (Figure 2b). Upon detailed morphological analysis, the senescent cells had a dramatic increase in the mitochondrial volume per cell (Figure 2c). This was largely driven by an increase in mitochondrial number (Figure 2d), rather than an increase in the size of individual mitochondria (Figure 2e). Despite the increase in cell volume and mitochondrial volume, there was no difference in the constitution of the mitochondria as assessed by blotting for the components of the electron transport chain normalized to total protein (Figure 2f and Figure S1h).

### 3.3 Intact oxidative phosphorylation in senescent fibroblasts

**FIGURE 3:**
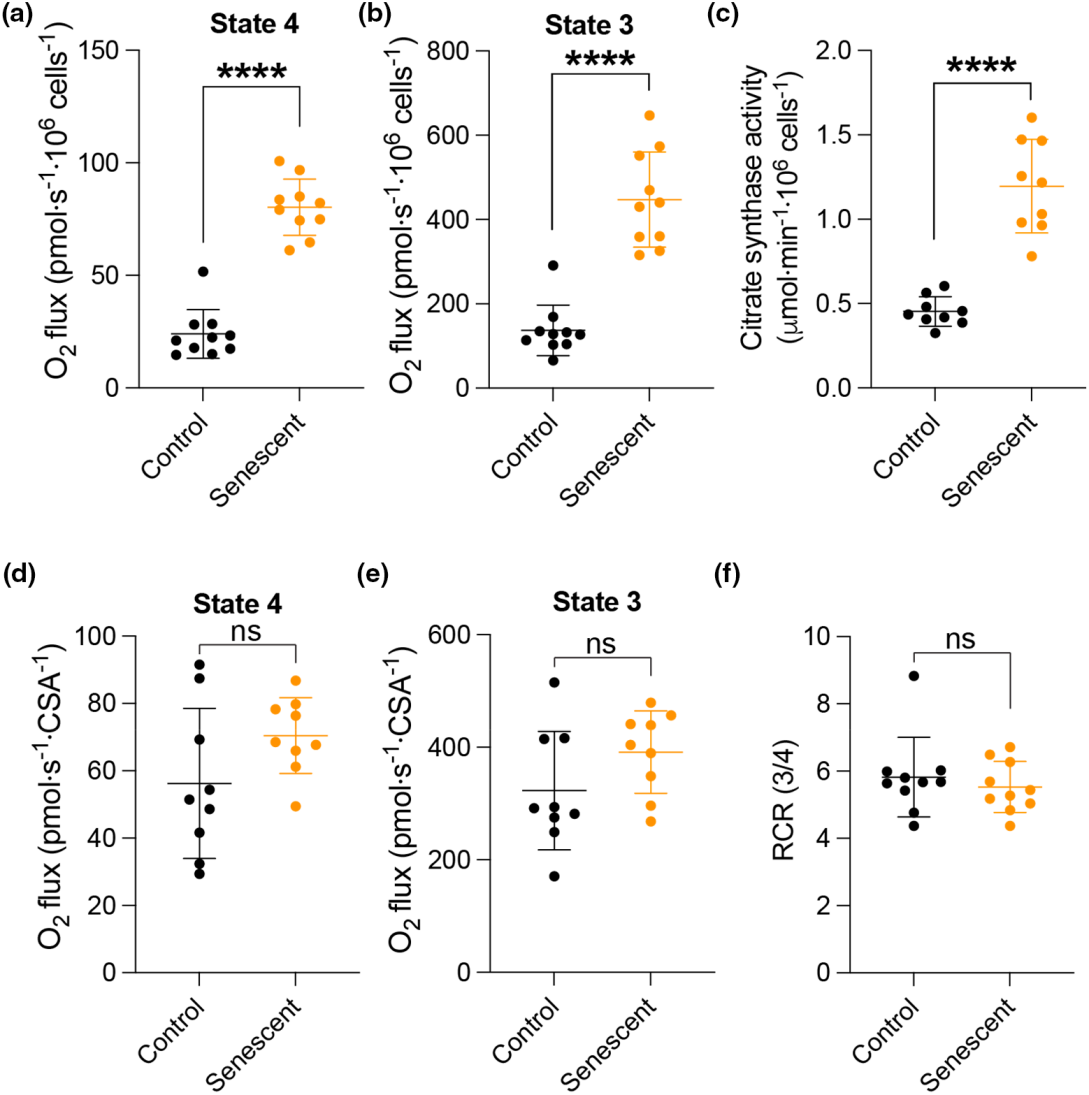
Mitochondrial respiratory capacity is increased due to changes in mitochondrial content in in vitro senescent fibroblasts. (a) State 4 respiration was measured using permeabilized iMEFs in the presence of pyruvate (5 mM), malate (2 mM), and glutamate (10 mM) normalized per million cells. (b) State 3 respiration measured as in (a) with the addition of 2 mM ADP. (c) Citrate synthase activity normalized per million cells. (d) State 4 respiration measured as in (a) normalized to citrate synthase activity. (e) State 3 respiration measured as in (b) normalized to citrate synthase activity. (f) Respiratory control ratio (RCR) or state 3 divided by state 4 is shown in (a‐b). Data shown are the mean ± SD for *n* = 9–10 per group. Data analyzed by Student's *t* test. ****p < 0.0001, ns, not significant.

We next examined mitochondrial function in senescent iMEFs. Following induction of senescence, we collected both control and senescent cells and evaluated mitochondrial respiratory capacity in permeabilized cells. Leak or state 4 respiration measured in the presence of metabolic substrates pyruvate, malate, and glutamate, but not ADP, was markedly elevated in senescent compared with control cells (Figure 3a). State 3 respiration, stimulated by the addition of ADP, reflects oxidative phosphorylation capacity and was similarly increased in senescent cells (Figure 3b). To determine whether the increased respiratory capacity in senescent cells reflected changes in mitochondrial function or mitochondrial content, we measured and normalized respiration rates to citrate synthase activity, which serves as a surrogate of mitochondrial mass (Hutter et al., 2004; Renner et al., 2003). Consistent with mitochondrial DNA copy number and content measurements (Figure 2), citrate synthase activity was significantly increased in senescent cells (Figure 3c). As a result, normalized state 4 and 3 respiratory rates were similar between groups (Figure 3d,e). Furthermore, the respiratory control ratio (state 3/state 4 or RCR), which is a simple and robust readout of mitochondrial function (Brand & Nicholls, 2011), was nearly identical for control and senescent cells (Figure 3f). Taken together, these findings implicate an increase in mitochondrial mass per cell and not an increase in function as the primary driver of increased respiratory capacity in senescent iMEF cells.

### 3.4 Mitochondrial gene expression does not change in response to telomere dysfunction in vivo

**FIGURE 4:**
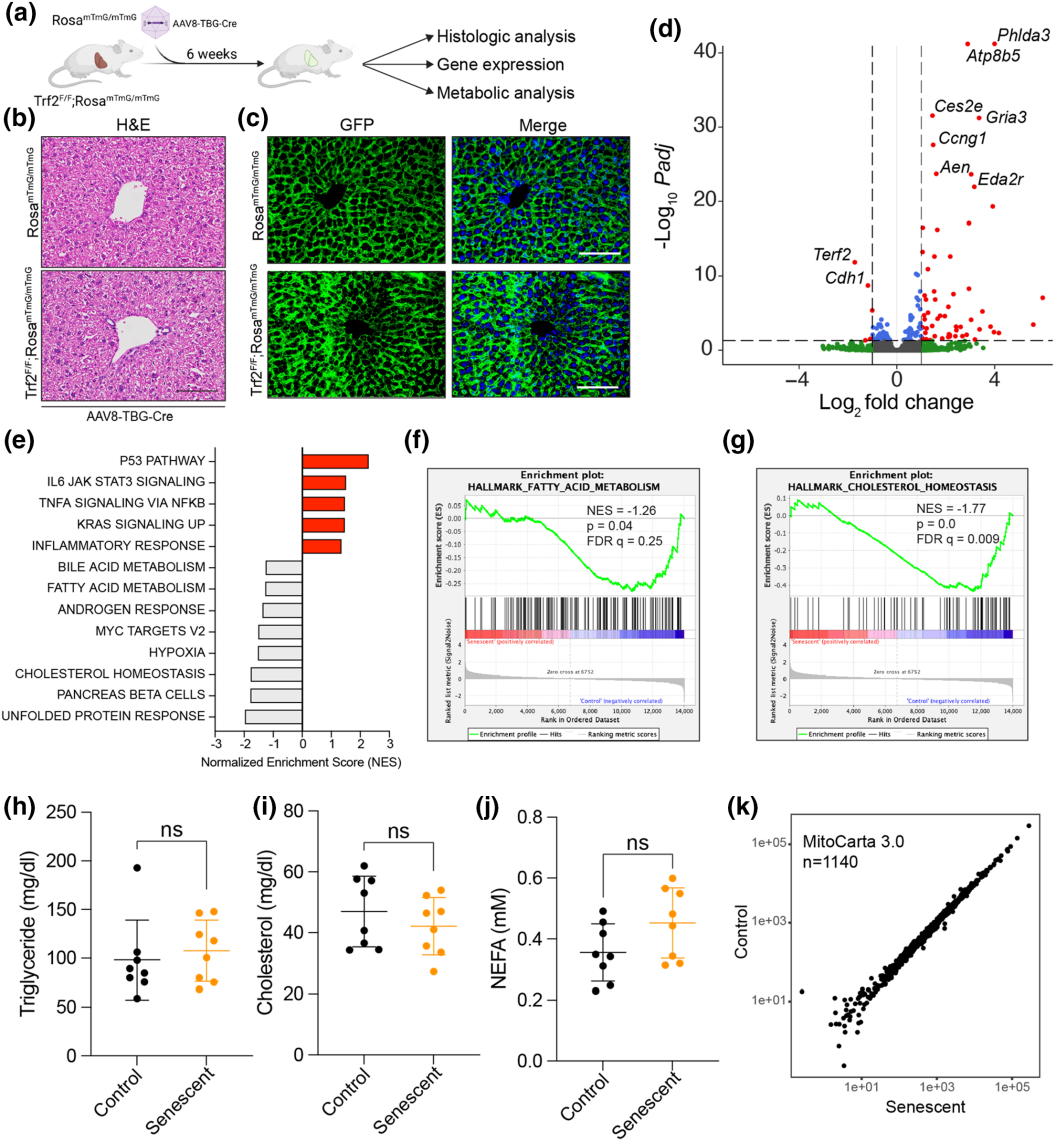
In vivo induction of hepatic telomere dysfunction. (a) Animal model of liver‐trophic viral infection leading to Trf2 deletion in experimental animals. The following analyses of these animals were done 6 weeks after AAV8‐TBG‐Cre viral infection. (b) Murine liver showing no changes in hepatic architecture. (c) Imaging of liver cryosections showing efficient activation of the GFP‐reporter. (d) Bulk RNA‐seq of senescent versus control murine liver homogenates reveals activation of *p53* pathway genes and downregulation of *Terf2*. (e) GSEA identified several hallmark pathways including p53 signaling, multiple inflammatory pathways, bile acid and fatty acid metabolism, and cholesterol homeostasis. (f, g) Enrichment plot for GSEA Hallmark Fatty Acid Metabolism and Cholesterol Homeostasis Pathways. (h–j) Blood lipid measurements in control versus senescent animals. Mean ± SD is shown, *n* = 8/group. Data analyzed by Student's *t*‐test. (k) Differential expression of MitoCarta 3.0 genes shows little change in senescence. Normalized counts from DESeq2 are shown. ns, not significant.

We reasoned that our iMEF findings might be distorted by immortalization and ex vivo culturing. To generate additional evidence to support our in vitro data, we next examined the mitochondrial consequences of telomere dysfunction in vivo. We generated a murine model of hepatocyte‐specific senescence using adenovirus‐delivered Cre recombinase and biallelic Trf2^F/F^ mice (Figure 4a). A liver‐focused model was chosen given the significant existing literature on hepatic mitochondrial isolation and respiratory studies (Chance & Williams, 1955; Edmunds et al., 2020; Frezza et al., 2007; Hogeboom et al., 1948), ease of viral targeting (Russell et al., 2019; Xie et al., 2022), and known tolerance of TRF2 deletion in this tissue (Lazzerini Denchi et al., 2006). Additionally, as a highly metabolically active tissue, we reasoned that our ability to detect differences between control and senescent tissues would be greater.

Both control (Rosa^mTmG/mTmG^) and experimental (Trf2^F/F^;Rosa^mTmG/mTmG^) animals were infected via tail vein injection with adeno‐associated virus vector serotype 8 that had been modified to express Cre recombinase under the control of the thyroxin binding globulin promoter (AAV8‐TBG‐Cre) (Figure 4a). Animals were observed for 6 weeks, and no discernible behavioral or gross differences were noted. Six weeks after AAV8‐TBG‐Cre injection, animals were sacrificed, and livers were collected for histologic and molecular analysis. No significant changes in hepatic morphology were noted (Figure 4b). We estimated transduction efficiency to have been 100% based on universal hepatocyte GFP expression in both groups (Figure 4c). Bulk RNA‐seq of the murine livers supported deletion of *Terf2*, engagement of the p53 pathway, and significant enrichment of genes associated with senescence (Figure 4d,e and Figure S2a,d). In contrast to iMEFs, deletion of *Terf2* in the murine liver led to differential expression of just 133 transcripts (Padj <0.05), highlighting the divergent responses to molecular stress in vitro and in vivo. Despite this, ~50% of the differentially expressed genes were also differentially expressed in the senescent iMEF dataset (64/133; Figure S2e). Gene set enrichment analysis suggested an upregulation in transcripts associated with the p53, Il6/Jak/Stat3 signaling, Tnfα signaling via Nfkβ, Kras signaling, and inflammatory response pathways (Figure 4e). In contrast, gene sets for fatty acid metabolism and cholesterol homeostasis both had negative enrichment scores that reached statistical significance (Figure 4f,g). To assess the in vivo relevance of these potential changes to lipid metabolism, we measured triglyceride, cholesterol, and non‐esterified nonfatty acid (NEFA) levels in plasma 6 weeks after transduction (Figure 4h–j). No significant differences were found. As in our iMEF data (Figure 1h), very few mitochondrial‐associated transcripts were differentially expressed in our bulk RNA‐seq dataset, and GSEA analysis of mitochondrial biogenesis showed no significant changes (Figure 4k and Figure S2b).

### 3.5 Mitochondrial function is intact in senescent hepatocytes in vivo

**FIGURE 5:**
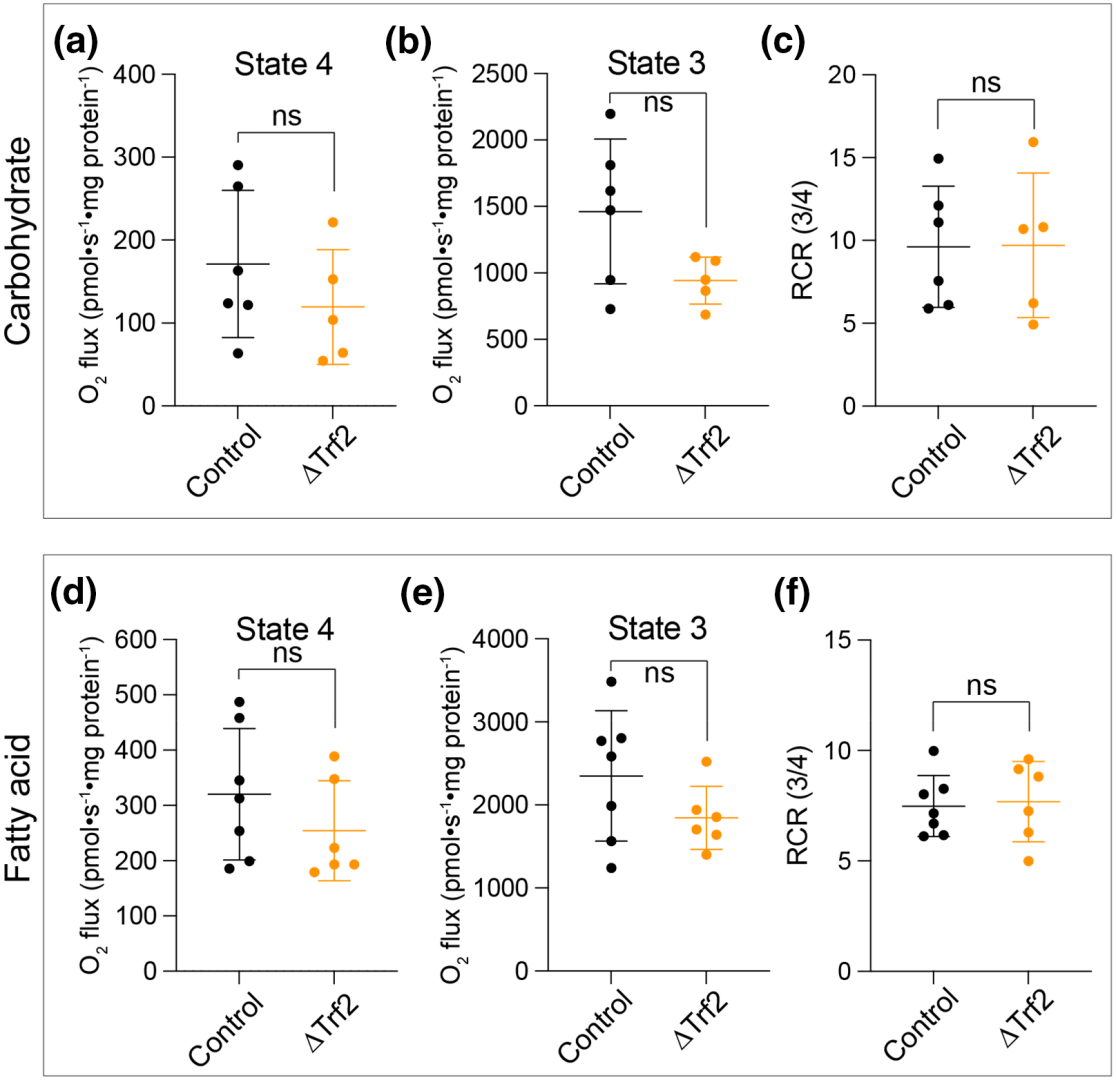
Mitochondrial respiratory capacity of the murine liver is intact despite telomere‐induced senescence. (a) State 4 respiration was measured using purified liver mitochondria in the presence of pyruvate (5 mM), malate (2 mM), and glutamate (10 mM) normalized per mg mitochondrial protein. (b) State 3 respiration was measured as in A with the addition of 2 mM ADP. (c) Respiratory control ratio (RCR) or state 3 divided by state 4 for data in a‐b. (d) State 4 respiration was measured using purified liver mitochondria in the presence of palmitoylcarnitine (0.025 mM), malate (2 mM), and glutamate (10 mM) normalized per mg mitochondrial protein. (e) State 3 respiration was measured as in (a) with the addition of 2 mM ADP. (f) RCR or state 3 divided by state 4 for data in d‐e. Data shown are the mean ± SD for *n* = 5–6 per group. Data analyzed by Student's *t* test, ns, not significant.

We next evaluated mitochondrial respiratory capacity in senescent and nonsenescent murine liver samples. Fresh liver samples were collected from the same animals described above and mitochondrial function was assessed in purified mitochondria (Figure 5), and mitochondrially enriched liver lysates (Figure S3) independently. We examined liver mitochondrial oxygen consumption rates in the presence of carbohydrate‐based substrates (pyruvate) (as we had done with iMEFs) as well as fatty acid‐based substrates (palmitoylcarnitine), the preferred substrate of liver hepatocytes (Alves et al., 2011). Similar to observations made in senescent iMEFs, state 4 and state 3 mitochondrial respiratory capacity in the presence of pyruvate or palmitoylcarnitine was unaffected by senescence and similar between control and hepatocyte‐specific conditional *Terf2* knockouts (Figure S3a–f). In contrast to senescent iMEFs, we observed no effect of *Terf2* deletion on liver citrate synthase activity (Figure S3g). Calculation of the RCR further demonstrated that induction of senescence via *Terf2* deletion did not affect mitochondrial respiratory function (Figure S3c,f). To ensure that our measurements were not influenced by subtle differences in mitochondrial content unaccounted for by citrate synthase activity, we repeated our analysis using isolated liver mitochondria from the same mice. Similar to the analysis of mitochondrially enriched liver homogenates, there were no significant changes in state 4, state 3, or RCR when comparing control and senescent liver mitochondria (Figure 5a–f).

## 4 DISCUSSION

We employed a tractable system for measuring the cellular and metabolic response to uniform telomere dysfunction in isolated cells in vitro and in vivo (Lazzerini Denchi et al., 2006). We found that mitochondrial‐associated transcripts and function were remarkably preserved following the induction of dysfunctional telomere‐mediated senescence in vitro and in vivo. This finding is consistent with the importance of mitochondria in supporting senescence‐related cellular functions (i.e., SASP) (Correia‐Melo et al., 2016). As entering senescence is a fate decision that potentially prevents the loss of cells that serve essential functions, it is plausible that cells would preserve mitochondrial activity given its necessity for maintaining cellular activities.

In contrast to our expectations, we found increased mitochondrial respiratory capacity in senescent iMEFs. The increased metabolic activity on a per‐cell basis is likely related to the significant increase in cell size and the compensatory increase in mitochondrial mass (Hutter et al. 2004). This is supported by our imaging analysis, mitochondrial:nuclear genome copy number measurement, and transcriptional analysis. When normalized to total protein or citrate synthase activity, the differences between control and senescent cells were abolished. Furthermore, the RCR, a robust measure of overall mitochondrial function that integrates the key features of oxidative phosphorylation (substrate oxidation, electron transport chain activity, ATP turnover, and uncoupling), was not different between control and senescent cells. Importantly, cell size and mitochondrial mass differences were noted in fibroblasts grown in culture, but not primary hepatocytes in vivo. This is likely due to the proliferative stimulus of cells grown in vitro compared to in vivo. When hepatocytes that lack TRF2 are challenged by partial hepatectomy, nuclear and cellular size increases (Lazzerini Denchi et al., 2006), suggesting that entrance to the cell cycle is required for cells to enlarge, even in the context of potent inducers of senescence. Taken together, our data support that senescent cells maintain mitochondrial/cytoplasmic ratios and mitochondria retain normal functional capacity even in disparate cell cycle conditions.

Our results conflict with a previous report of telomere dysfunction disrupting mitochondrial biogenesis in mice (Sahin et al., 2011). Preceding investigations of mitochondrial function had evaluated late‐generation telomerase knockout mice with short telomeres. Laboratory mice have extremely long and heterogenous telomeres, and telomere dysfunction in mice lacking telomerase components is variable (Blasco et al., 1997). Telomere shortening likely influences tissues at different times due to differences in replicative histories, and given the paracrine consequences of telomere dysfunction; it is possible that systemic sequelae could be caused by telomere dysfunction in any organ system. Furthermore, given that mice with short telomeres have mucosal blunting of the gastrointestinal tract (Armanios et al., 2009), it is possible that defects in nutrient absorption could contribute to a reduction in mitochondrial activity. Our approach was distinct in that we drove acute telomere dysfunction as opposed to gradual telomere shortening, and it is possible that this approach accounts for some of the differences in our findings. However, analysis of transcriptional profiling data from human fibroblasts entering replicative senescence also revealed few changes in the expression of mitochondrial‐associated genes, similar to our findings. Moreover, a recent report found increased glucose utilization and fatty acid oxidation in cells entering replicative senescence, supporting that these pathways are preserved or more active in senescence (Chan et al., 2022). While there are clear differences between telomere uncapping and telomere shortening, it appears that mitochondrial function is preserved in both settings.

We identified substantial differences in the response to the same senescence stimulus in vitro versus in vivo. While mitochondrial function was intact, iMEFs exhibited widespread transcriptional changes, with more than ~20% of annotated genes being differentially expressed. In contrast, the same senescence stimulus drove far fewer transcriptional changes in primary hepatocytes in vivo (only ~0.5% of genes were differentially expressed). Despite the difference in the number of differentially expressed genes, both cell types shared a core set of 64 genes that were differentially expressed in both cell types (Figure S2d). This list includes several previously described senescence‐associated genes including *Cdkn1a*, *Gdf15*, and *Mdm2*, but also many novel transcripts that appear to be unique to this study. Our findings highlight a significant challenge in understanding the response to senescence—transcriptional and secretory behavior are sensitive to both the cause of senescence and the specific cell type affected (Basisty et al., 2020). Our results also draw attention to the risk of studying senescence in vitro, where artificial conditions can contribute to the observed response to pro‐senescent stimuli.

Our findings suggest that mitochondrial activity is preserved in response to dysfunctional telomere‐mediated senescence. Our study is limited by the analysis of only two distinct cell types and their responses at a single time point. Alternative cell types may respond differently to telomere dysfunction, including the possibility that mitochondrial biogenesis or function may be disrupted in those contexts. We purposely selected hepatocytes due to their metabolic complexity—including their ability to engage in both anaplerotic and cataplerotic metabolic flux and their dependence on mitochondrial activity to maintain cellular function. We reasoned that this would be a rigorous system to evaluate the consequences of senescence on mitochondrial content and function. As we did not include longitudinal analysis, it is possible that the response to telomere dysfunction may change over time as cells adapt. Indeed, mitochondrial quality control by mitophagy declines in senescent cells (Dalle Pezze et al., 2014; García‐Prat et al., 2016) such that senescence‐associated mitochondrial dysfunction may be a later developing consequence of the senescent phenotype. Future studies will likely require evaluating the response to multiple pro‐senescence stimuli on multiple cell types in vivo and at distinct time points. However, given the selective maintenance of mitochondrial function observed here, even after substantial genetic perturbation, it appears that evolution has selected for the preservation of metabolic activity after entrance to cellular senescence.

## AUTHOR CONTRIBUTIONS

DIS, ALM, MK, BAK, MJJ, and JKA conceptualized the project and planned experiments. DIS, FMB, AGS, KMR, LG, and KEL performed experiments and processed data. DIS, MJJ, and JKA analyzed data and prepared figures. JKA and DIS drafted the manuscript, and all co‐authors edited and approved the manuscript.

## CONFLICT OF INTEREST STATEMENT

The authors declare no competing interests.

## Supporting information


Appendix S1–S3.

## Data Availability

All data generated in this study have been deposited in the appropriate public database or are available upon request. All raw transcriptional profiling data are available at NCBI's Gene Expression Omnibus GSE226613.
