# Supplementary material for: Intact mitochondrial function in the setting of telomere‐induced senescence
Source: Aging Cell. 2023 Sep 8;22(10):e13941. doi: 10.1111/acel.13941 (PMC10577573; doi:10.1111/acel.13941)
Supplement: Supplementary file 1 — Appendix S1–S3. [file ACEL-22-e13941-s001.docx]

**Intact mitochondrial function in the setting of telomere-induced senescence**

Daniel I. Sullivan^1^, Fiona M. Bello^2,3^, Agustin Gil Silva^1^, Kevin M. Redding^3,4^, Luca Giordano^3,4^, Angela M. Hinchie^1^, Kelly E. Loughridge^1^, Ana L. Mora^5^, Melanie Königshoff^1^, Brett A. Kaufman^3,4^, Michael J. Jurczak^2,3^*, Jonathan K. Alder^1^*

^1^Dorothy P. and Richard P. Simmons Center for Interstitial Lung Disease, Division of Pulmonary, Allergy, and Critical Care Medicine, University of Pittsburgh; Pittsburgh, PA, USA.

^2^Division of Endocrinology and Metabolism, University of Pittsburgh; Pittsburgh, PA, USA.

^3^Center for Metabolism and Mitochondrial Medicine, University of Pittsburgh; Pittsburgh, PA, USA.

^4^Heart, Lung, and Blood Vascular Medicine Institute, University of Pittsburgh; Pittsburgh, PA, USA.

^5^Division of Pulmonary, Critical Care and Sleep Medicine, Davis Heart Lung Research Institute, The Ohio State University, Columbus, OH, USA.

*Corresponding Authors. Email: [jalder@pitt.edu](mailto:jalder@pitt.edu) and jurczakm@pitt.edu

**Supplemental Materials**

**
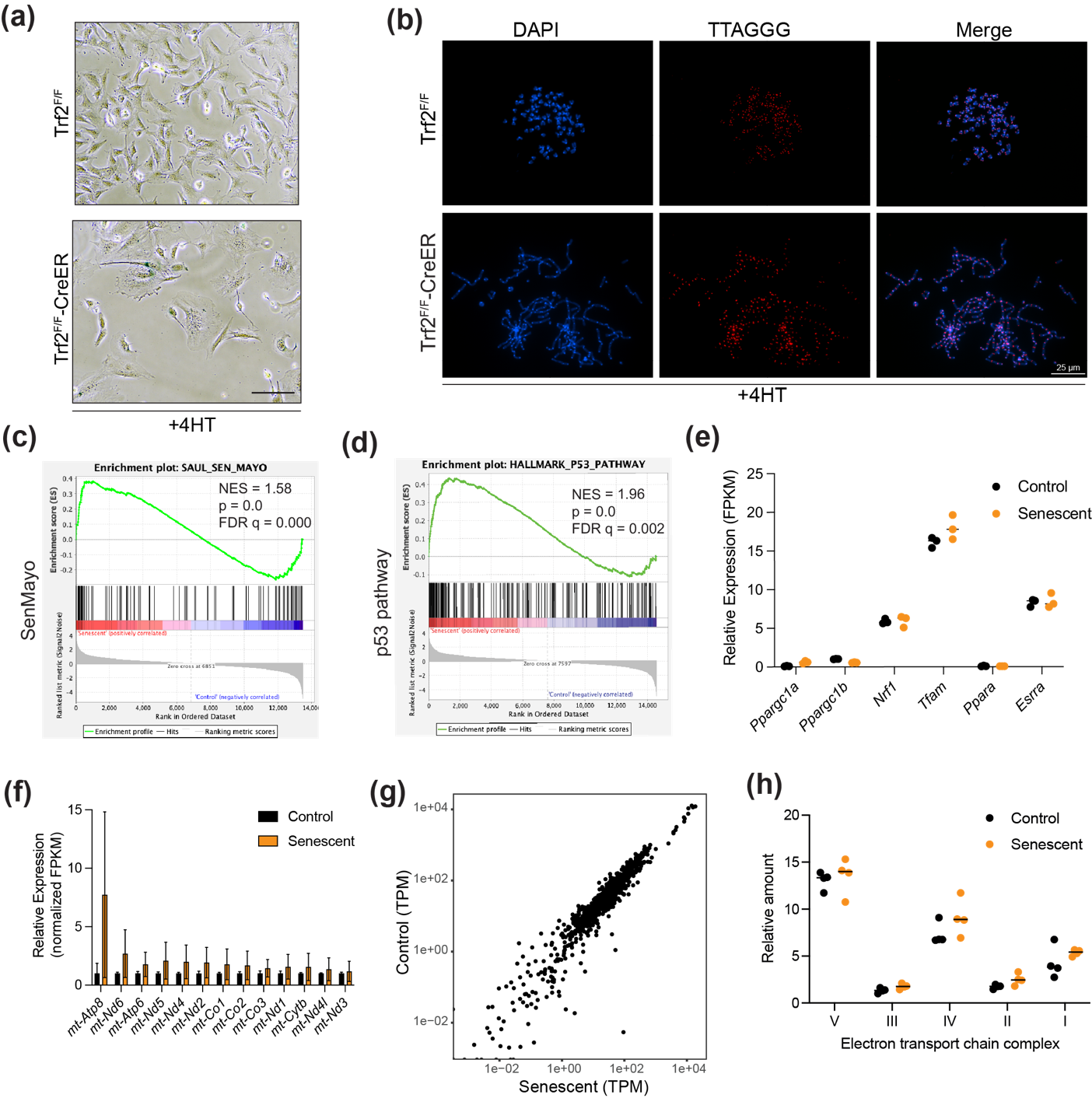
**

**Supplemental Figure 1. Characterization of murine fibroblast cell line following the induction of dysfunctional telomere-induced senescence.** (a) Phase contrast photomicrograms of iMEFs seven days after addition of tamoxifen stained for senescence associated beta galactosidase. (b) Representative photomicrographs of metaphase spreads and fluorescence in situ hybridization of control iMEFs (Trf2^F/F^) and senescent iMEFs (Trf2^F/F^-CreER) seven days after the addition of 4-hydroxy tamoxifen (4HT). DNA is stained with DAPI (blue). Telomeres are stained red using a Cy3 conjugated TTAGGG PNA probe. Scale bar is 25 microns. (c) Enrichment plot for the GSEA SenMayo gene set. (d) Enrichment plot for GSEA Hallmark p53 Pathway for the iMEF RNA-Seq dataset. (e) Relative expression of mitochondria-associated genes in our iMEF RNA-Seq dataset that were previously reported to be downregulated in response to short telomeres. FPKM = fragments per kilobase of transcript per million mapped reads. (f) Transcripts encoded by the mitochondrial genome from the iMEF RNA-Seq dataset. (g) Differential expression of MitoCarta 3.0 genes from human fibroblasts (WI-38) showing similar expression in control and cells entering replicative senescence (from GSE175533). (h) Quantification of mitochondrial electron transport chain proteins from western blot of control and senescent iMEF lysates shown in Figure 2f. ***P < 0.001, Mann-Whitney test.


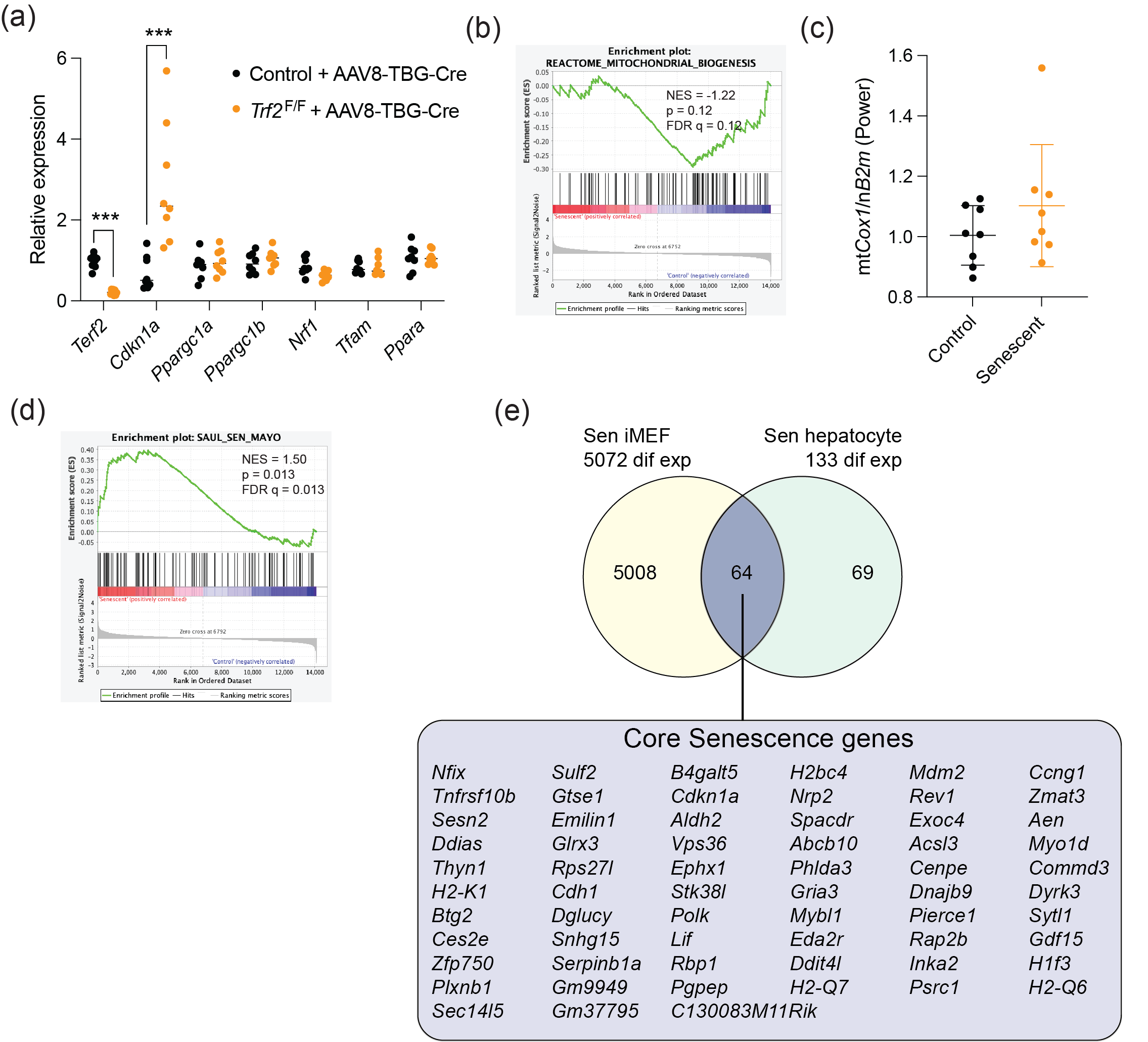


**Supplemental Figure 2. Intact mitochondrial biogenesis in senescent hepatocytes.** (a) Quantitative PCR of transcripts from control and senescent murine livers 6-7 weeks after AAV8-TBG-Cre infection. (b) Enrichment plot for Reactome Mitochondrial Biogenesis Pathway for the senescent hepatocytes RNA-Seq dataset. (c) Mitochondrial:nuclear DNA ratio for control vs senescent murine liver tissue. Mean ± SD is shown, n=8/group. (d) Enrichment plot for the GSEA SenMayo gene set showing strong enrichment of senescence associated genes in hepatocytes *in vivo*. (e) Gene expression overlap between senescent iMEFs and senescent murine hepatocytes in the setting of telomere-mediated senescence.


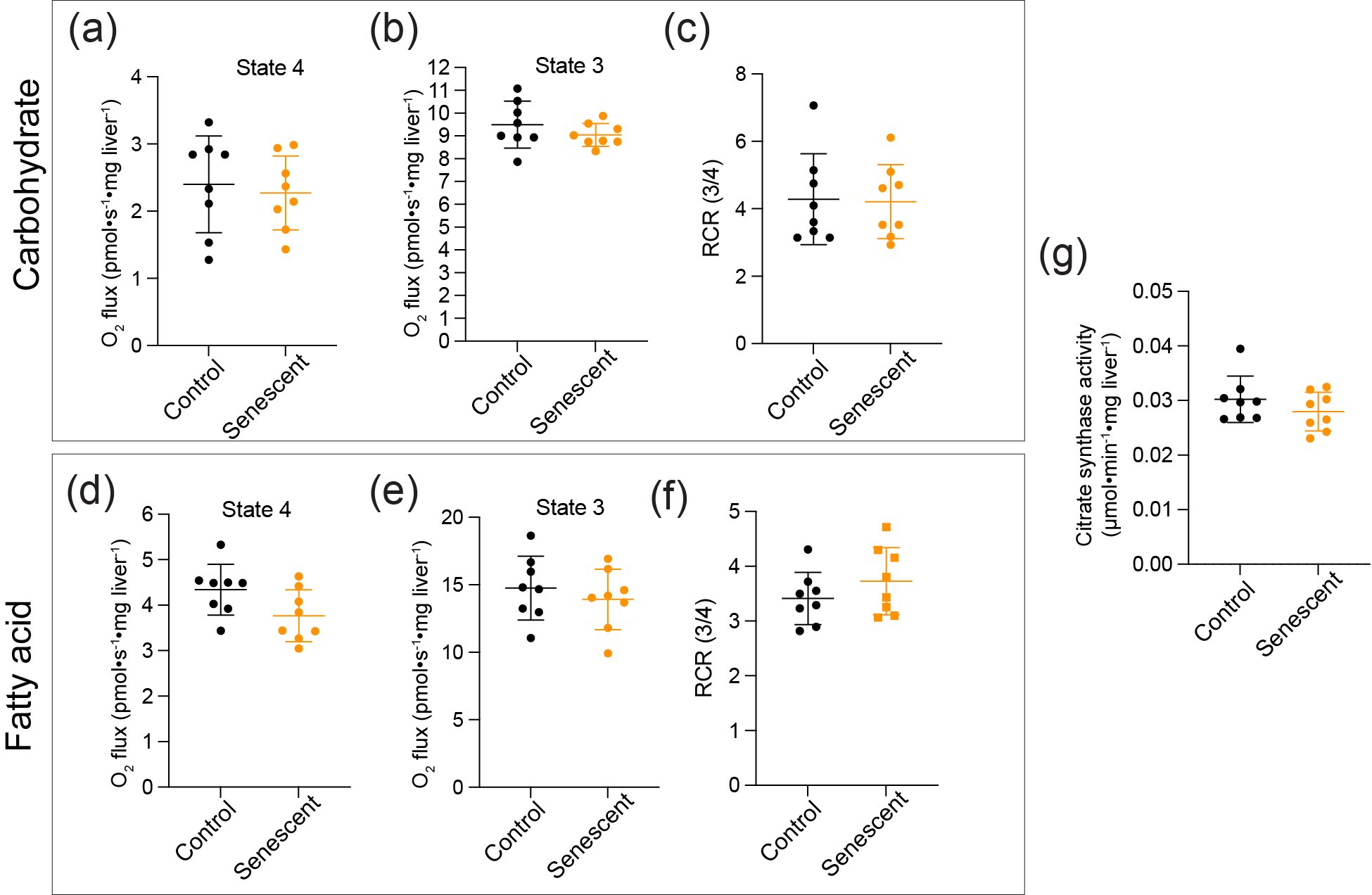


**Supplemental Figure 3. Mitochondrial respiratory capacity of mitochondrially-enriched liver lysates in the setting of telomere-induced senescence.** Upper panel is carbohydrate substrate. Lower panel is fatty acid substrate. (a,d) State 4 respiration measured using post-nuclear supernatants from liver homogenates made in a mitochondrial isolation buffer, assayed in the presence of pyruvate (5 mM), malate (2 mM), and glutamate (10 mM) normalized per mg liver in each sample. (b,e) State 3 respiration measured as in (a) with the addition of 2 mM ADP. (c,f) Respiratory control ratio (RCR) or State 3 divided by State 4 for each of the respective substrates. (g) Citrate synthase activity normalized per mg liver in each sample. Data shown are the mean ± SD for n=8 per group. Data analyzed by Student’s t-test.
